# Hypouricemic Effects of Extracts From *Agrocybe aegerita* on Hyperuricemia Mice and Virtual Prediction of Bioactives by Molecular Docking

**DOI:** 10.3389/fphar.2018.00498

**Published:** 2018-05-15

**Authors:** Tianqiao Yong, Shaodan Chen, Yizhen Xie, Ou Shuai, Xiangmin Li, Diling Chen, Jiyan Su, Chunwei Jiao, Yalei Liang

**Affiliations:** ^1^State Key Laboratory of Applied Microbiology Southern China, Guangdong Provincial Key Laboratory of Microbial Culture Collection and Application and Guangdong Open Laboratory of Applied Microbiology, Guangdong Institute of Microbiology, Guangzhou, China; ^2^Guangdong Yuewei Edible Fungi Technology Co., Guangzhou, China

**Keywords:** *Agrocybe aegerita*, hyperuricemia, xanthine oxidase, bioactives, virtual screening

## Abstract

*Agrocybe aegerita* has long been utilized for promoting diuresis in traditional Chinese medicine (TCM) with a close correlation to hypouricemia. Ethanol (AAE) and water (AAW) extracts of the compound led to a remarkable decrease in serum uric acid levels (SUA) in hyperuricemia mice, approaching that of the normal control. Both AAE and AAW exhibited suppression effects on hepatic xanthine oxidase (XOD) activities and elevation effects on renal OAT1 (organic anion transporter 1). However, only little negative impact was observed on the inner organ functions. The molecular docking was used to screen our in-home compound database for *A. aegerita*, and four compounds including 2-formyl-3,5-dihydroxybenzyl acetate, 2,4-dihydroxy-6-methylbenzaldehyde, 2-(6-hydroxy-1H-indol-3-yl)acetamide, and 6-hydroxy-1H-indole-3-carbaldehyde (HHC) were identified as potential active compounds. Their inhibitory mechanism on XOD might be attributed to their localization in the tunnel for the entrance of substrates to XOD active site, preventing the entrance of the substrates. To confirm the activity of the screened compounds experimentally, HHC was selected due to its high ranking and availability. The assaying result suggested the significant inhibitory activity of HHC on XOD. Also, these compounds were predicted to carry good ADME (absorption, distribution, metabolism, and excretion) properties, thereby necessitating further investigation. The current results provided an insight into the hypouricemic effects of macrofungi and their bioactives, which might provide the significant theoretical foundation for identifying and designing novel hypouricemia compounds.

## Introduction

Macrofungi has been utilized as foods, nutraceuticals, and medicines around the world for a number of diseases due to their unique tastes and health benefits (Diyabalanage et al., 2008). Among these, the edible mushroom *Agrocybe aegerita* is an agaric fungus that has a wide-range of growth distribution and is commercially cultured in Asia, North America, and Europe. The abundant protein content (25–30% in weight) provides luxury nutrition daily for humans (Petrovic et al., 2015). Moreover, polysaccharides with hypoglycemic activity (Kiho et al., 1994), indole derivatives (Kim et al., 1997), sesquiterpenoids (Aoki and Ohta, 2010) with free radical scavenging activity, protein bound-polysaccharide (Hyun et al., 1996) with antitumor activity, alkaloids (Koshino et al., 1996), sterols suppressing the formation of osteoclast (Choi et al., 2009), and many antibiotic, anti-fungal constituents (Ngai et al., 2005; Zhu et al., 2010) were discovered in *A. aegerita*. Importantly, it has been exploited by Asian folks, especially Chinese, as an herbal medicine, for strengthening the spleen functions and promoting diuresis for hundreds of years, which associate with hyperuricemia; thus, we hypothesis that it might affect hyperuricemia.

Hyperuricemia refers to the level of SUA (serum uric acid) > 6.2 mg/dL that can directly cause gout and kidney stones. It also accelerates the cardiovascular and renal diseases (Harris et al., 1999; Shin et al., 2011; Rock et al., 2013), thereby exhibiting a prevalent risk to human life. This high SUA, effectuated by enhanced synthesis of uric acid in the liver or by declined excretion through impaired renal function, crystallizes as monosodium urate needles that further induce inflammation repeatedly (Saag and Choi, 2006). Therefore, lowering the levels of SUA against hyperuricemia and gout is essential. In this respect, the first treatment was to use uricosurics; for example, benzbromarone and lesinurad enhance the uric acid excretion through the kidney by interacting with ion transporters, especially URAT1 (uric acid transporter 1). However, these molecules might be restricted by hypersensitive and allergic reactions (Harrold, 2013). The second method was to reduce uric acid production by suppressing xanthine oxidase (XOD), which oxidizes xanthine to uric acid (Cao et al., 2011). Although allopurinol and febuxostat are the major XOD inhibitors, they are limited by low efficacy (40% for allopurinol), Stevens-Johnson syndrome (Halevy et al., 2008), and cardiovascular complications (Becker et al., 2010). Thus, studies exploring the nutraceuticals of low toxicity and high efficiency are under intensive focus.

Herein, we investigated the hypouricemia effects of *A. aegerita* and virtual screening of the putative active compounds by computational technology. First, we prepared ethanol (AAE) and water (AAW) extracts of *A. aegerita*, followed by assaying their effects on SUA and UUA (urine uric acid). In order to identify the underlying mechanisms, hepatic XOD and renal transporters were examined by enzyme-linked immunosorbent assay (ELISA) and Western blotting. Also, their effects on renal function were evaluated by assaying blood urea nitrogen (BUN) and creatinine. Additionally, the inner organ coefficients were recorded for assessing their effects on inner organs. In order to identify the potential bioactives from *A. aegerita*, molecular docking screening using the in-home compound database for *A. aegerita* was conducted, and the top ranked compounds were identified from *A. aegerita* as potential active compounds. Experimentally, the available compound was also tested. Additionally, their ADME properties were predicted.

## Experimental

### Materials

Adamas Reagent Co. (Shanghai, China) supplied potassium oxalate (PO), hypoxanthine (HX), allopurinol, and benzbromarone. Uric Acid Kits were obtained from Nanjing Jian-Cheng Bioengineering Institute (Nanjing, China). URAT1 and XOD Elisa Kits were provided by R&D System Inc. (Minneapolis, USA). *A. aegerita* samples were identified by Guangdong Yuewei Edible Fungi Technology Co. (Guangzhou, China) in October 2016 and obtained for the present study. In the herbarium of Guangdong Institute of Microbiology, a voucher specimen (YW20161009-AA) was preserved.

### Preparation and characterization of AAE and AAW

*A. aegerita* fruiting body (100 g) was submerged 3 times in ethanol at 65°C for to obtain AAE (4.15 g, 4.15%). The remains were extracted with water 3 times to obtain the AAW (7.13 g, 7.13%). The HPLC fingerprints (Figures S1, S2) of AAE and AAW and standard chemical are described in Supporting Information, where the three characteristic peaks were identical and overlapped well.

### Animals

The experiment was designed and conducted by/at the Guangdong Institute of Microbiology (GT-IACUC201612011). Male SPF Kunming mice (20 ± 2 g) were obtained from the Guangdong Provincial Medical Laboratory Animal Centre (Guangzhou, China). The method reported by our group previously (Yong et al., 2016) was utilized for establishing the hyperuricemia model by dose 600 mg/kg HX and 100 mg/kg PO. Allopurinol and benzbromarone were used as positive controls.

### Drug administration

Mice were divided into normal, hyperuricemia, allopurinol, and benzbromarone controls and drug groups (*n* = 10) with AAE and AAW at various doses, respectively. The mice of allopurinol and benzbromarone controls were administered allopurinol (5 mg/kg) and benzbromarone (7.8 mg/kg). For AAE and AAW, the mice were orally administered 50, 100, and 200 mg/kg each, respectively.

### Evaluations of uric acid, XOD, URAT1, OAT1, BUN, and creatinine

Serum and urine were collected for uric acid (Carroll et al., 1971), BUN (Talke and Schubert, 1965), BUN and creatinine measurements. Uric acid was examined based on the phosphotungstic acid reaction. BUN was measured based on the urease reaction. Creatinine was determined spectrophotometrically using the Jaffe reaction method.

Livers and kidneys were homogenized to determine XOD and URAT1 by ELISA according manufacture's protocols, respectively. This method utilizes the quantitative sandwich enzyme immunoassay technique. A specific XOD/URAT1 monoclonal antibody was pre-coated onto a 96-well microplate. Standards, control, and samples were pipetted and XOD/URAT1 was bound by the pre-coated antibody. After washing any unbound substances clearly, a specific XOD/URAT1 enzyme-linked polyclonal antibody was added. Following washing, a substrate solution was added. After incubation, the reaction mixture was stopped by stop solution. The intensity of the color measured is in proportion to the amount of mouse XOD/URAT1 bound in the initial step. The sample values are then read off the standard curve.

URAT1 and OAT1 (organic anion transporter 1) were also evaluated by Western blotting. Immunoblotting was assayed using URAT1 (1: 2000; Rabbit URAT1 Antibody, 14937-1-AP, ProteinTech Group, Chicago, USA) and OAT1 (1:2000; OAT1 Antibody, ab135924, Abcam Inc., Cambridge, USA) as well as GAPDH (1: 4000; GAPDH Antibody, GB13002-m-1, Servicebio Co., Wuhan, China) antibodies. The contents of target proteins were analyzed via densitometry using Alpha Innotech (AlphaEaseShop, USA) and normalized by the respective blotting from GAPDH.

### Organ coefficient and body weight growth

After the experiment, the inner organs were gathered and weighed and the inner organ coefficients recorded as organ weight/body weight. The body weight growth was recorded as percentages of increase using day 1 as 100% (body weight growth = day n/day 1 × 100%).

### Statistical analysis

The obtained data were treated by one-way analysis of variance (ANOVA) in SPSS and demonstrated as mean ± standard error. The difference was set at *P* < 0.05 or *P* < 0.01 as compared by two-tailed Student's *t*-test.

### Virtual screening by molecular dockings

The molecular docking screening was conducted using CDOCKER (Wu et al., 2003), which was a CHARMm-based tool with a rigid receptor. XOD crystal structure (PDB ID, 1FIQ) was set as the receptor, in which salicylate was selected as the active site and then removed (Enroth et al., 2000). The molecules of *A. aegerita* were collected from literature and their 3D structures illustrated and energetically minimized using the CHARMm force field. Docking was executed using the default parameters to obtain poses that were refined using the annealing molecular dynamics. For final poses, the CHARMm energy (-CDOCKER_ENERGY, interaction energy plus ligand strain) and the binding energy (-CDOCKER_INTERACTION_ENERGY) were computed. Poses with the highest -CDOCKER_ENERGY were selected for further analysis.

### XOD inhibition assay

The XOD inhibitory activity by screened compounds was examined sepectrophotometrically at 290 nm by a Multimode Microplate reader (Thermo Scientific, USA). After incubation of XOD (50 μL, 7.5 × 10^−8^ mol L^−1^ in PBS with pH at 7.5) with compounds (50 μL in PBS with pH at 7.5) at various concentration for 15 min in a 96-well microplate, xanthine (150 μL, 5.0 × 10^−5^ mol L^−1^ in PBS with pH at 7.5) was added. Then, the reaction process was monitored and recorded by the Multimode Microplate reader. Every reaction was repeated 5 times. PBS and allopurinol were used as negative and positive control. The relative activity (%) = (slope of reaction kinetics equation obtained by reaction with inhibitor)/(slope of reaction kinetics equation obtained by reaction without inhibitor) × 100.

### ADME property prediction

Blood-brain barrier penetration (BBB), human intestinal absorption (HIA), and CYP2D6 inhibition were assessed with the Discovery Studio 3.5 (Accelrys Inc., San Diego, USA). Of these, CYP2D6 participates in the metabolism of various substrates in the liver and its suppression involves several drug-drug interactions, thereby rendering the prediction of ADME as essential. The similarity of the compounds to the drug was also assessed following Lipinski's rule of five (Lipinski, 2004).

## Results

**Figure 1:**
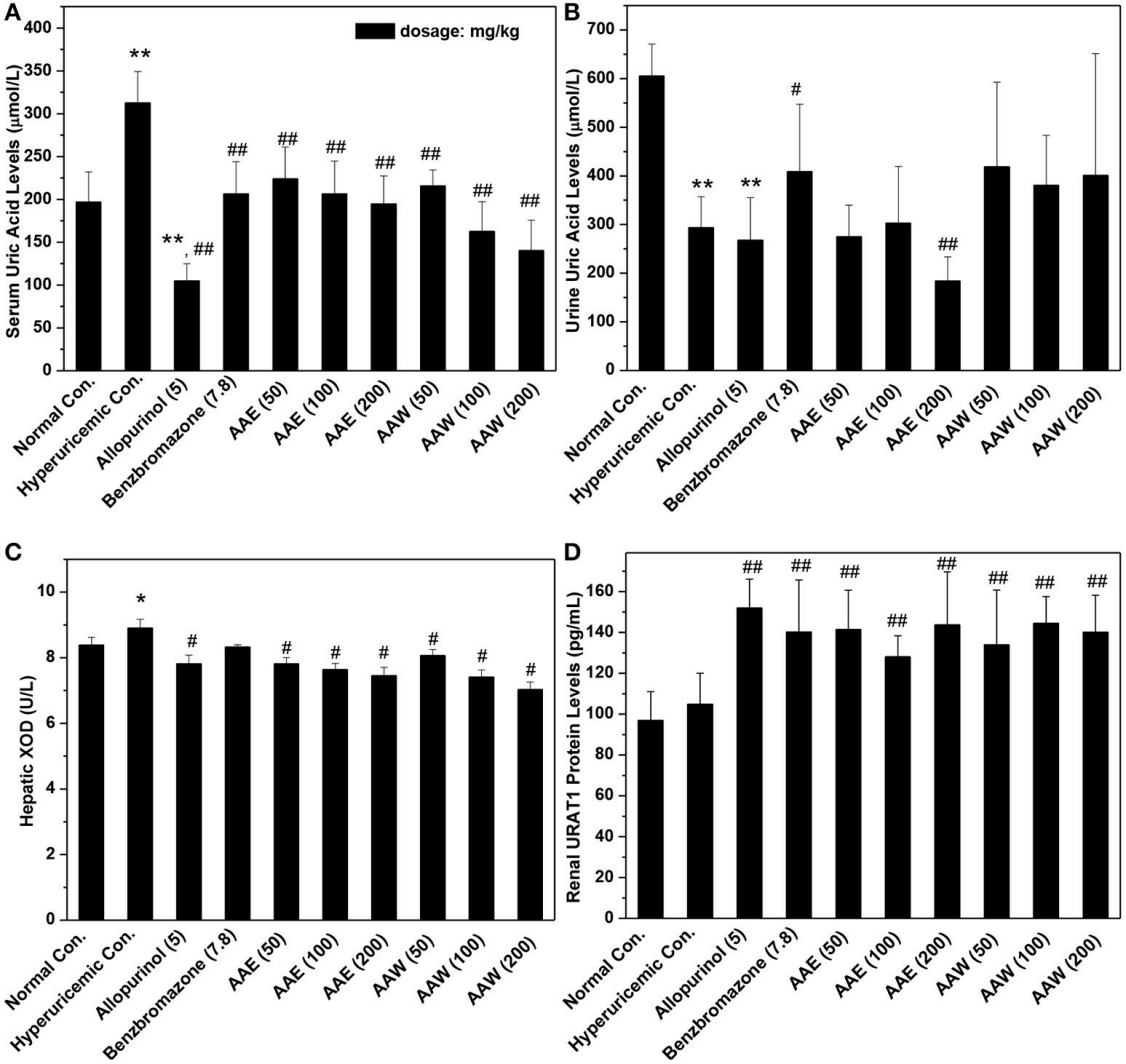
Actions of AAE and AAW on **(A)** SUA, **(B)** UUA, **(C)** XOD, and **(D)** URAT1 (*n* = 10). Uric acid was examined based on the phosphotungstic acid reaction. XOD activity and URAT1 protein were tested by Elisa method according to manufacturer's protocols. ^*^P < 0.05 or ^**^P < 0.01 *vs*. normal control; ^#^*P* < 0.05 or ^##^*P* < 0.01 *vs*. hyperuricemic control.

The hypouricemia activities of AAE and AAW were assessed by assaying the level of SUA in hyperuricemia mice (Figure 1A). The models were established successfully by crystal elevated SUA from 196 μmol/L in normal control to 312 μmol/L, *P* < 0.01 in the hyperuricemia control. Allopurinol (5 mg/kg) decreased the level of SUA of hyperuricemia mice to 104 μmol/L (*P* < 0.01), which established the model from another perspective. Another positive drug benzbromarone (7.8 mg/kg) also reduced the level of SUA to 206 μmol/L (*P* < 0.01), similar to that of the normal control (196 μmol/L). Notably, AAE and AAW gave rise to remarkable hypouricemia activities, which lowered the level of SUA to 223, 206, and 194 μmol/L (*P* < 0.01) for AAE at 50, 100, and 200 mg/kg and 215, 162, and 140 μmol/L (*P* < 0.01) for AAW at 50, 100, and 200 mg/kg in hyperuricemia mice. Similar to benzbromarone, these SUA of AAE and AAW groups approached normal control.

In addition, the effects on UUA were examined (Figure 1B). In the hyperuricemia control, PO reduced UUA (293 μmol/L, *P* < 0.01) as compared to the normal control (605 μmol/L). Furthermore, allopurinol further reduced the level of UUA in hyperuricemia animals (267 μmol/L). However, benzbromarone at 7.8 mg/kg enhanced the UUA level in hyperuricemia mice (408 μmol/L, *P* < 0.05). AAE at 50 and 100 mg/kg (except 184 μmol/L for 200 mg/kg) did not exhibit a significant efficiency in promoting UUA in hyperuricemia mice (275 and 302 μmol/L, respectively). AAW at 50, 100, and 200 mg/kg did not exhibit a similar efficacy (418, 380, and 401 μmol/L).

The effects of AAE and AAW against hepatic XOD were examined (Figure 1C). The effect of XOD activities in the hyperuricemia control (8.91 U/L) was higher than that in normal control (8.38 U/L, *P* < 0.05). As expected, allopurinol was lowered to 7.81 U/L (*P* < 0.05), which was lower than the normal control. Importantly, AAE at 50, 100, 200 mg/kg reduced XOD activity to 7.81, 7.64, and 7.45 U/L (*P* < 0.05), respectively, showing obvious inhibitory effects on XOD. In addition, AAW at 50, 100, 200 mg/kg reduced that to 8.06, 7.40, and 7.02 U/L (*P* < 0.05) respectively, showing similar inhibitory effects on XOD. Thus, XOD suppression may be one of the mechanisms underlying hypouricemia actions of *A. aegerita*.

The effects of AAE and AAW on renal URAT1 were primarily analyzed by ELISA (Figure 1D). No significant difference was noted between the normal control group (96.95 pg/mL) and hyperuricemia group (104.85 pg/mL). Compared to the hyperuricemia group, renal URAT1 protein levels of allopurinol groups (151.96 pg/mL, *P* < 0.01) were elevated significantly. The URAT1 protein levels in the AAE and AAW treatment groups were 141.50, 128.12, and 143.67 for AAE at various doses and 133.96, 144.49, and 140.17 pg/mL for AAW at different doses, higher than the hyperuricemia control (*P* < 0.01).

**Figure 2:**
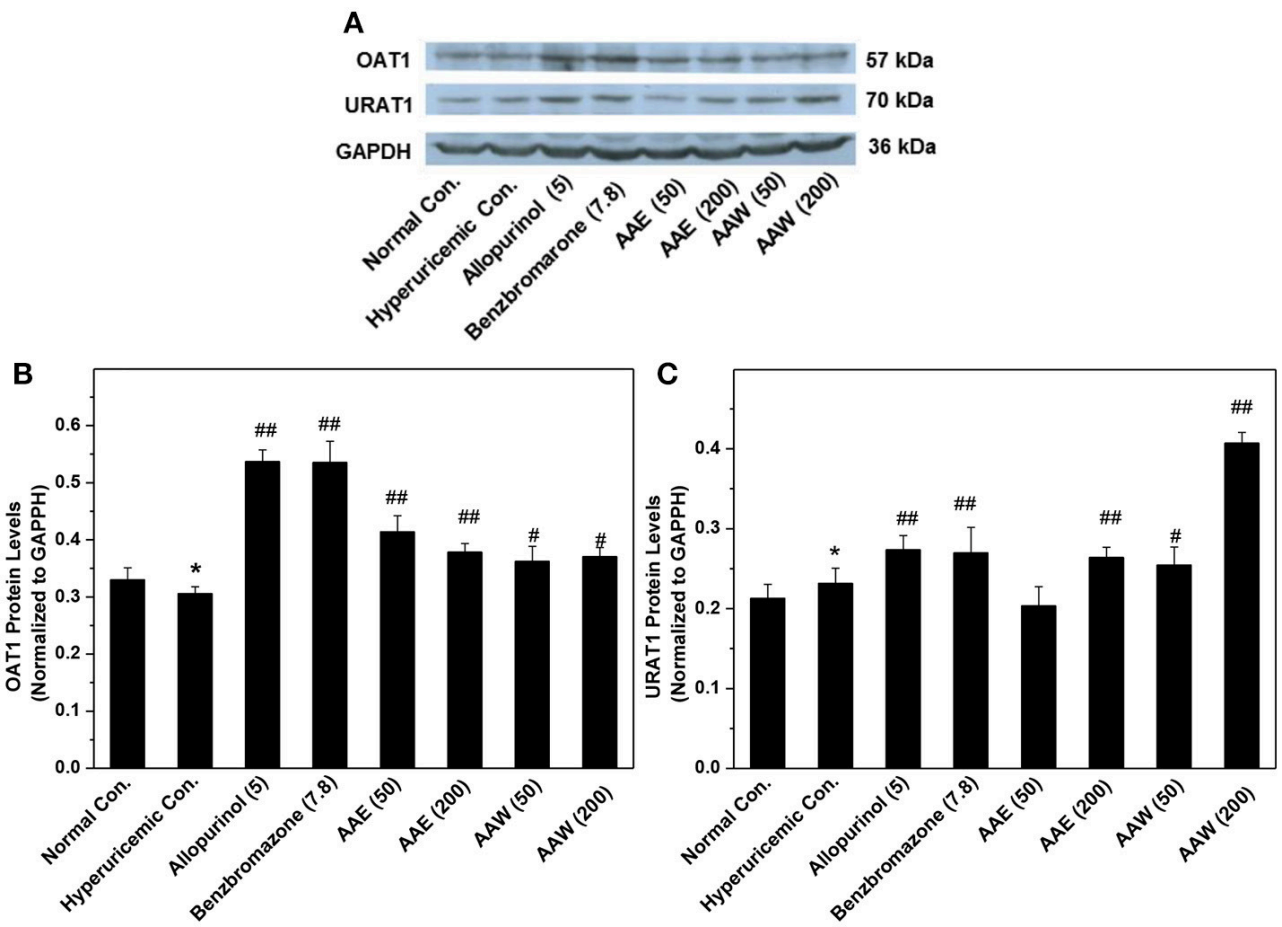
Western blot analysis (*n* = 3) of renal OAT1 **(A,B)** and URAT1 **(A,C)**. Immunoblotting was assayed using URAT1 (1:2000) and OAT1 (1:2000) as well as GAPDH (1: 4000) antibodies. The contents of target proteins were analyzed via densitometry using Alpha Innotech (AlphaEaseShop, USA) and normalized by the respective blotting from GAPDH. ^*^P < 0.05 *vs*. normal control; ^#^*P* < 0.05 or ^##^*P* < 0.01 *vs*. hyperuricemic control.

Also, two major transporters in the kidney were analyzed by Western blotting (Figure 2), which demonstrated that AAE (except AAE at low dose) and AAW increased the level of OAT1 and URAT1. This phenomenon was in agreement with the results of ELISA.

**Figure 3:**
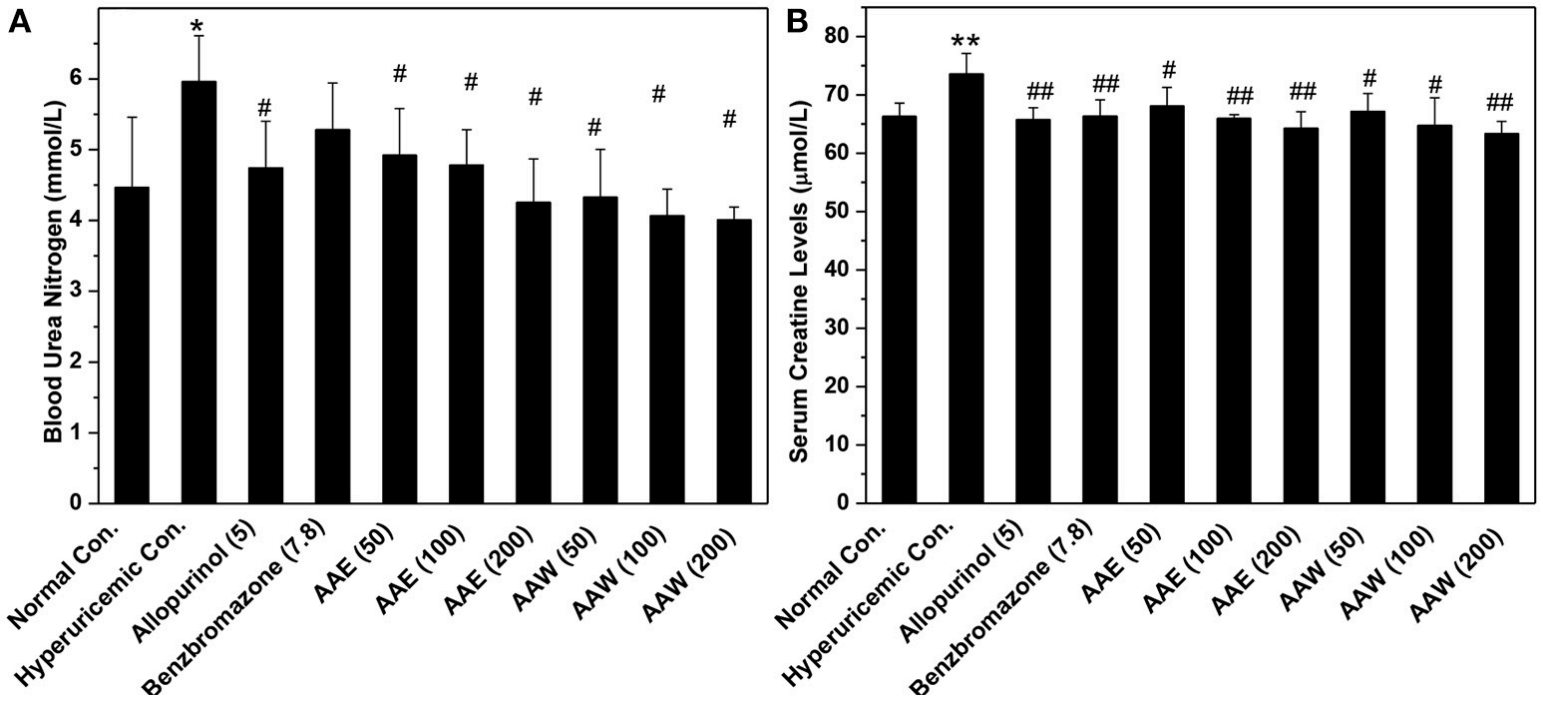
Influences of AAE and AAW on **(A)** BUN and **(B)** creatinine (*n* = 10). BUN was measured based on the urease reaction. Creatinine was determined spectrophotometrically using the Jaffe reaction method. ^*^P < 0.05 or ^**^P < 0.01 *vs*. the normal control; ^#^*P* < 0.05 or ^##^*P* < 0.01 *vs*. hyperuricemic control.

The hyperuricemia control exhibited BUN at 5.96 mmol/L, which was higher than that of the normal control (4.47 mmol/L, *P* < 0.05, Figure 3A). BUN in the allopurinol group (4.74 mmol/L) was slightly lower than that in the hyperuricemia group. The groups with *A. aegerita* decreased the BUN levels to 4.92, 4.78, and 4.25 mmol/L for AAE and 4.32, 4.06, and 4.00 for AAW at several doses (*P* < 0.05), respectively, similar to the normal control. The hyperuricemia control exhibited a serum creatinine level of 73.54 μmol/L, which was higher than that of the normal control (66.27 μmol/L, Figure 3B). *A. aegerita* (*P* < 0.05) reduced it to 68.10, 65.92, and 64.27 for AAE and 67.15, 64.75, and 63.37 for AAW.

**Figure 4:**
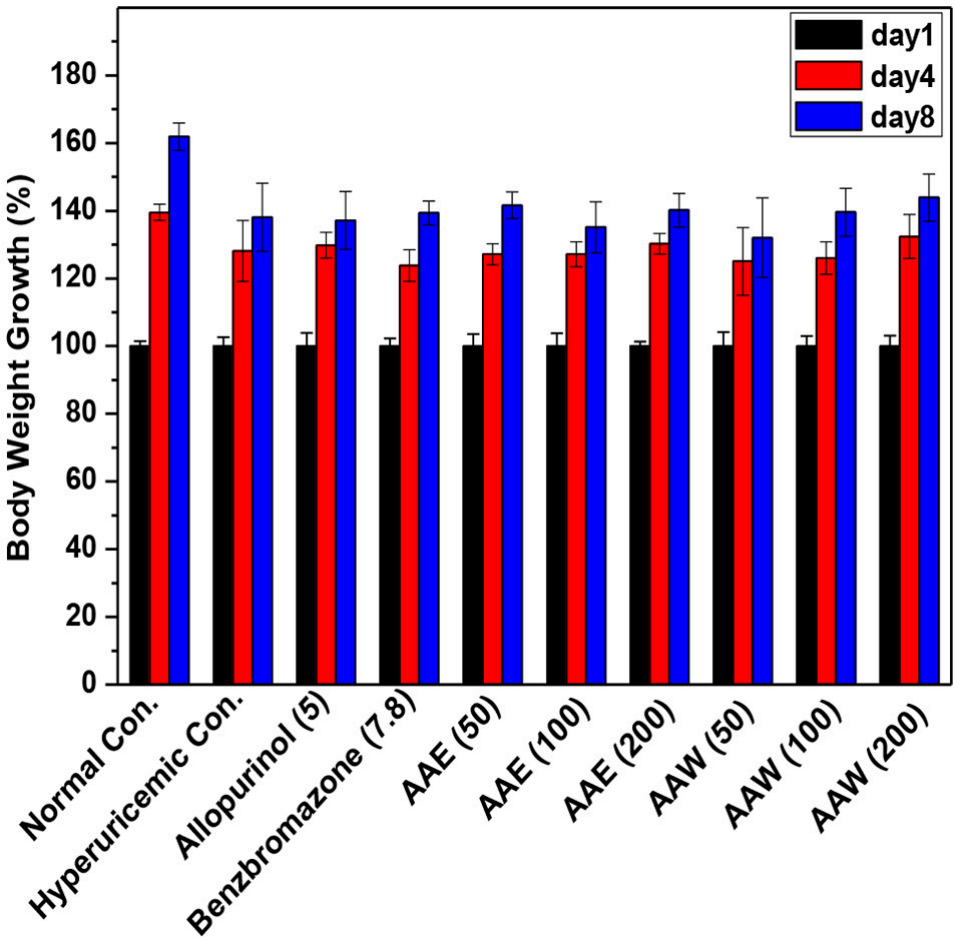
Affections of AAE and AAW on body weight growth (*n* = 10). The body weight growth was recorded as percentages of increase using day 1 as 100%.

On days 1, 4, and 8, the body weight growths of mice were recorded (Figure 4). The increase in the body weight of the model mice increased gradually, and similarity (*P* > 0.05) were analyzed between the drugged and hyperuricemia mice. In addition, all mice showed normal water and diet consumption and good mental state during the experiment.

**Figure 5:**
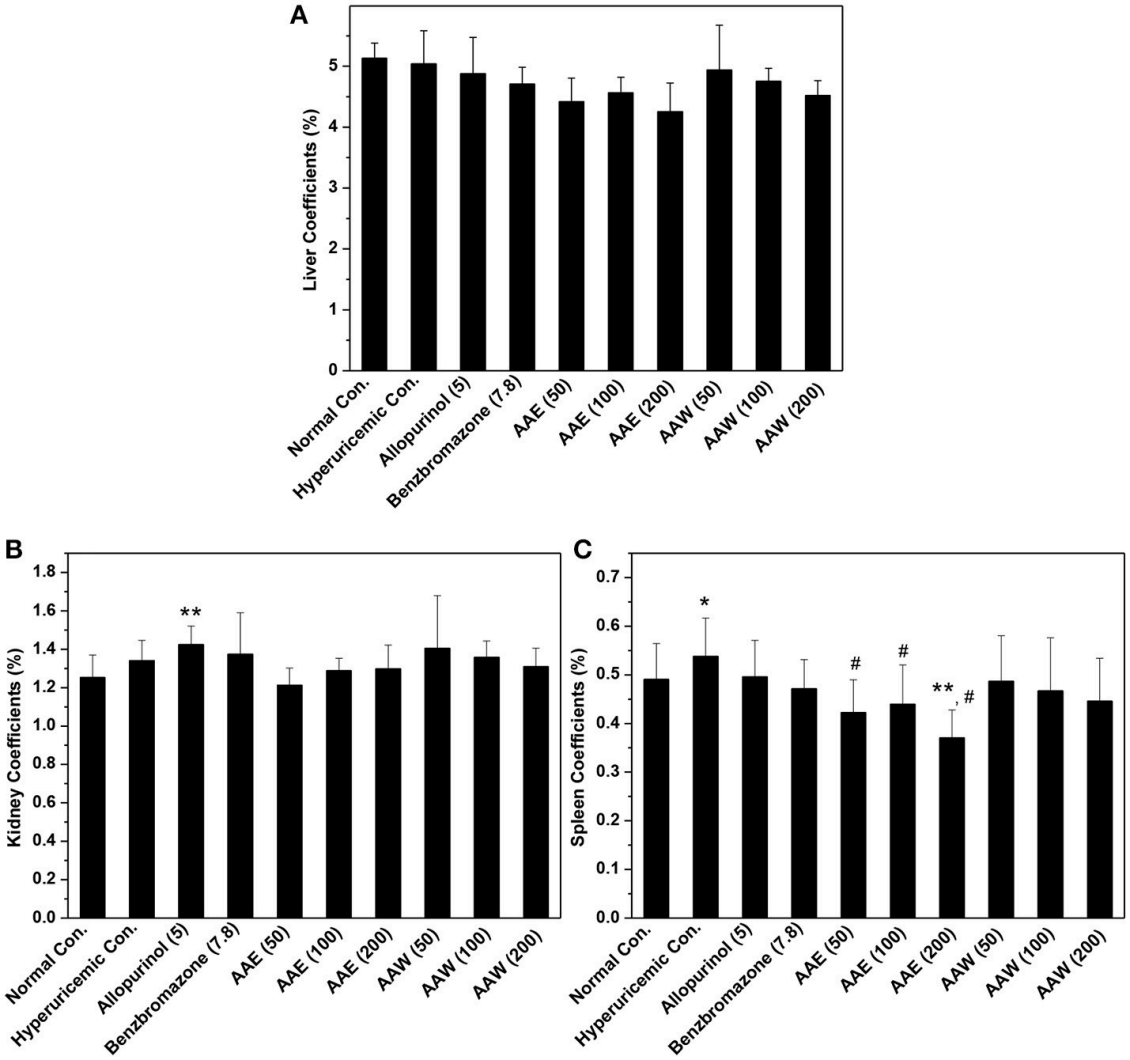
Affections of AAE and AAW on **(A)** liver, **(B)** kidney, and **(C)** spleen (*n* = 10). The inner organ coefficients were recorded as organ weight/body weight. ^*^P < 0.05 or ^**^P < 0.01 *vs*. normal control; ^#^*P* < 0.05 *vs*. hyperuricemic control.

The inner organ coefficients were recorded to examine the toxicity of *A. aegerita* (Figure 5). The liver coefficients for all controls and *A. aegerita* groups were similar, suggesting little impact by AAE and AAW. Apparently, allopurinol increased the kidney coefficients to 1.42% as compared to the normal control (1.25%, *P* < 0.01). Moreover, some swelling occurred in the allopurinol control. The kidney coefficients with AAE and AAW were 1.21, 1.28, and 1.29 for AAE at different doses and 1.40, 1.36, and 1.31 for AAW at different doses, correspondingly, similar to the normal control. With respect to the spleen, hyperuricemia control (0.53%) had a higher coefficient than normal control (0.49%, *P* < 0.05, Figure 5C). However, AAE at different doses lowered spleen coefficient to 0.42, 0.44, and 0.37% (*P* < 0.05), especially the high-dose caused an extreme decrease (*P* < 0.01). Also, AAW showed similar coefficients to the normal control.

**Figure 6:**
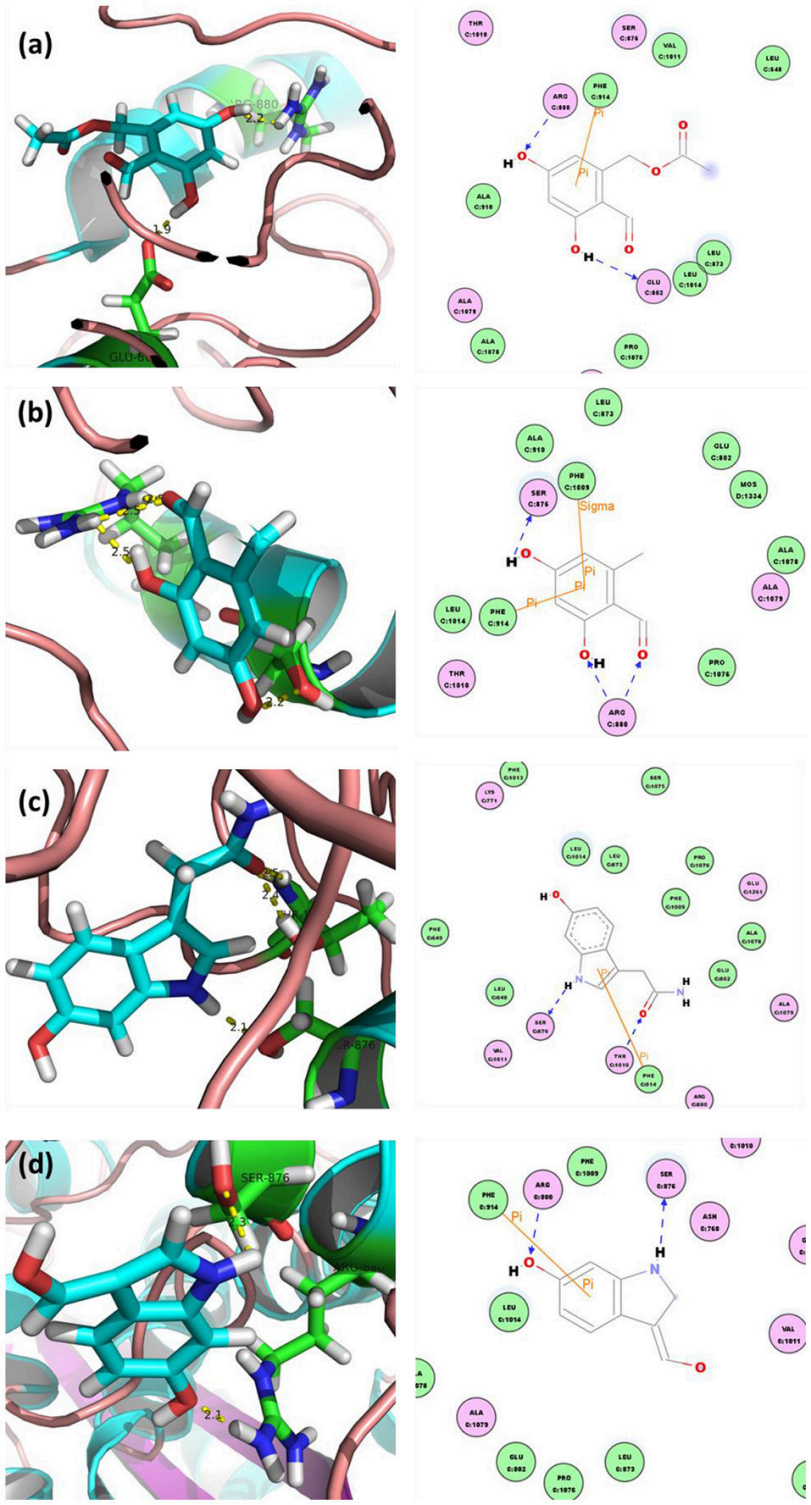
Docked complex of XOD with top ranked 4 compounds from *A. aegerita*: **(A)** XOD-2-formyl-3,5-dihydroxybenzyl acetate; **(B)** XOD-2,4-dihydroxy-6-methylbenzaldehyde; **(C)** XOD-2-(6-hydroxy-1H-indol-3-yl)acetamide; **(D)** XOD-6-hydroxy-1H-indole-3-carbaldehyde (HHC). Yellow dashed line sand blue lines represent hydrogen bonds; brown lines represent Pi-Pi and Pi-sigma interactions.

The in-house compound database for *A. aegerita* was screened by molecular docking, and the most energetically favorable binding candidates were selected. Among the library, 2-formyl-3,5-dihydroxybenzyl acetate, 2,4-dihydroxy-6-methylbenzaldehyde, 2-(6-hydroxy-1H-indol-3-yl)acetamide, and 6-hydroxy-1H-indole-3-carbaldehyde (HHC) were the top hits (Table 1). These compounds were located in the hydrophobic cavity (Figure 6) and interact with the molybdenum atomic (Mo) domain. Among them, 2-formyl-3,5-dihydroxybenzyl acetate had a CDOCKER_ENERGY and interaction energy of−26.7587 and−255.419 kcal/mol, respectively. In the interaction pattern, the residues, Glu802 and Arg880, participate in hydrogen (H) bonding (Figure 6A). Especially, Glu802 and Arg880 interact with the H atoms of two hydroxyls attached to the aromatic ring. PHE914 forms Pi-Pi bonding with the aromatic ring of the compound. 2,4-dihydroxy-6-methylbenzaldehyde had a CDOCKER_ENERGY and interaction energy at −25.9467 and −150.1420 kcal/mol. Arg880 and Ser876 are involved in the H bond interactions. Arg880 forms 2 H bonds with oxygen atoms belonging to the hydroxyl and aldehyde groups on the aromatic ring of the compound. Ser876 forms H bonds to H atoms owned by hydroxyls on the aromatic ring of the compound. Phe914 interacts with it through a Pi-Pi bond via the aromatic ring. Phe1009 interacts with it through a Sigma-Pi bond with the aromatic ring. 2-(6-Hydroxy-1H-indol-3-yl)acetamide had a CDOCKER_ENERGY and interaction energy of −23.3433 and −163.64157 kcal/mol. Ser876 forms an H bond with the H atom of the amine group in the bicyclic moiety. Thr1010 links the oxygen atom of acylamino group to the bicyclic moiety through H bonding. Moreover, Phe914 interacts with the compound through a Pi-Pi bond. 6-Hydroxy-1H-indole-3-carbaldehyde (HHC) presents a CDOCKER_ENERGY and interaction energy of −17.1663 and −133.116 kcal/mol. Ser876 forms an H bond with H atom of the amine group in the bicyclic moiety. Arg880 forms an H bond with H atom of the hydroxyl group in the aromatic moiety of the compound. Phr914 forms a Pi-Pi bond with the aromatic ring.

**Table 1:** Structures and ADME predictions of screened compounds.

**Compounds**	**Structure**	**HIA level*^a^***	**Solubility level*^b^***	**BBB level*^c^***	**CYP2D6 class**	**PPB class**	**Hepatotoxic class**	**Lipniski's filter**	**Ames prediction**
2-formyl-3,5-dihydroxybenzyl acetate	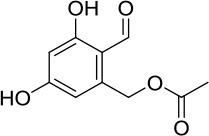	0	4	3	false	false	false	yes	non-mutagen
2,4-dihydroxy-6-methylbenzaldehyde	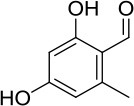	0	4	3	false	false	true	yes	non-mutagen
2-(6-hydroxy-1H-indol-3-yl)acetamide	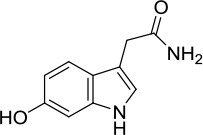	0	4	3	false	false	true	yes	non-mutagen
6-hydroxy-1H-indole-3-carbaldehyde	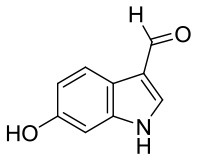	0	3	2	false	false	true	yes	non-mutagen

a 0 describes good absorption

b 3 depicts good and 4 optimal

c *2 demonstrates Brain-Blood ratio between 0.3:1 and 1:1 and 3Brain-Blood ratio less than 0.3:1*.

**Figure 7:**
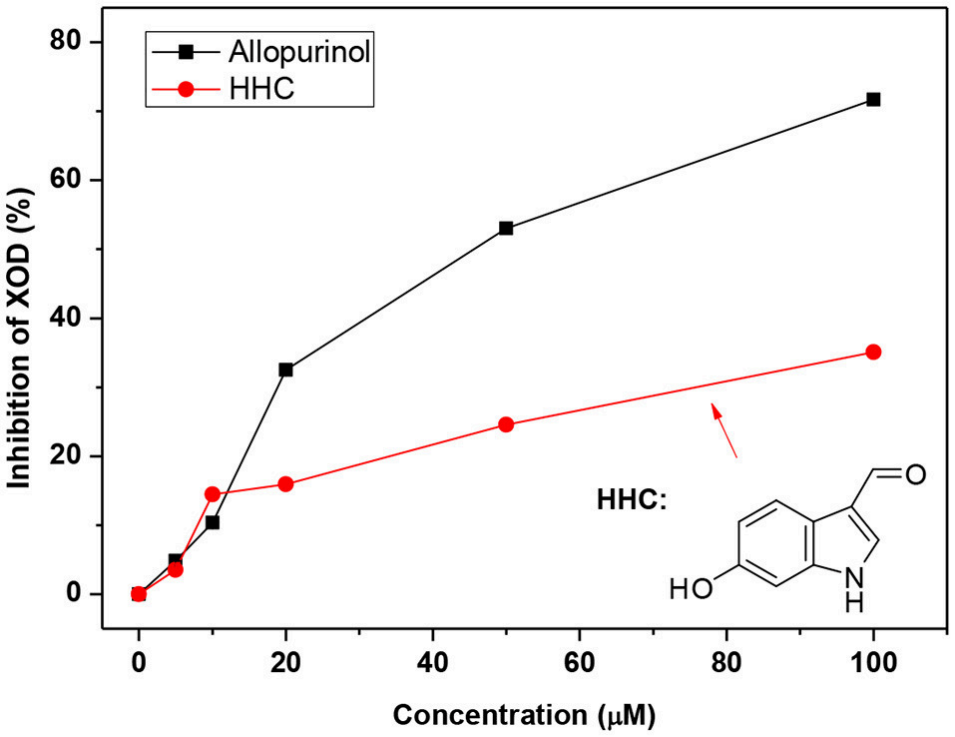
XOD inhibition in the presence of allopurinol and HHC at various concentrations (pH = 7.4, *T* = 298 K). c (XOD) = 7.5 × 10^−8^ mol L^−1^, and c (xanthine) = 5.0 × 10^−5^ mol L^−1^.

To test the compounds screened, we performed XOD inhibitory assaying using HHC due to its high ranking and availability and allopurinol as control. As shown in Figure 7, the activity of XOD was inhibited by HHC in a concentration-dependent manner. The enzymatic activity decreased to 50% of the initial level when the concentration of HHC (IC_50_) reached to [(240.92 ± 0.27) 10^−6^ mol L^−1^], which was some higher than that of allopurinol [(44.73 ± 0.27) 10^−6^ mol L^−1^]. The results indicated that HHC had ability to inhibit XOD.

For forecasting the bioavailability of the four compounds, their ADME properties were evaluated by exploiting the descriptors in the Discovery Studio (Table 1), where HIA, BBB, and CYP2D6 inhibition were involved. The compounds putatively exhibited good intestinal absorption (HIA level = 0), good or optimal solubility (solubility level = 3 or 4), and low BBB index (BBB level = 2 or 3). The four compounds were unable to inhibit CYP2D6 and bind with carrier proteins in the blood. Moreover, these four compounds followed the rule of Lipinski's filter.

## Discussion

Hyperuricemia and its related diseases are prevalent, especially gout (Liu et al., 2015), risking the human life globally. Therefore, following cancer, diabetes, and hyperlipidemia, hyperuricemia is under intensive focus. However, as commonly prescribed drugs, allopurinol and benzbromarone, are of limited use due to a series of side reactions (Pacher et al., 2006). Since the options for hyperuricemia treatment are limited, the current on-going studies are focused on the same. Presently, we described a natural macrofungus, *A. aegerita*, for ameliorating hyperuricemia.

The extracts AAE and AAW were prepared using water and ethanol, respectively as they are as safe and environment-friendly solvents (Meireles, 2009). Therefore, these are widely used in the food and drug manufacture industry. Herein, these were selected and used in ethanol followed by water, consequently according to the rules of safety and environment-friendliness.

Primarily, the models were vital for the functional foods or drug discovery. The elevated level of SUA in hyperuricemia mice suggested the further success of hyperuricemia model (Yong et al., 2016). The study modeled the occurrence of hyperuricemia in humans, which featured high-purine feeding and low excretion by the kidney. On the other hand, the significant reductions in the levels of SUA in allopurinol and benzbromarone further confirmed the success of the hyperuricemia mice model. Importantly, AAE and AAW produced remarkable hypouricemia effects on this model, showing crystal hypouricemia activities.

UUA plays a critical role in uric acid homeostasis of SUA, and hence, UUA was recorded. In the case of hyperuricemic control, PO serves as an uricase inhibitor as well as a barrier for electrogenic uric acid transporter/channel. Comprehensively, it elevated the level of UUA as compared to the normal control. The slight decrease in the UUA level in the allopurinol control in comparison with the hyperuricemia control may be attributed to the declines of uric acid synthesis induced by its inhibitory effect on XOD. As a commonly prescribed uricosuric, benzbromarone significantly elevated the level of UUA since its interaction with URAT1 in order to block the uric acid reabsorption by the kidney. Unlike benzbromarone, no elevations in the UUA level were observed for AAE and AAW in comparison with the hyperuricemia control. This might suggest that AAE and AAW did not significantly affect the suppression of uric acid reabsorption.

Due to the key role of XOD in purine metabolism and the influence on SUA (Enroth et al., 2000), we evaluated the effects of AAE and AAW on XOD. As expected, allopurinol reduced the level of hepatic XOD lower than that of the normal control, which was in agreement with previous studies (Pacher et al., 2006). Accordingly, the AAE and AAW acted similarly on XOD to allopurinol, suggesting critical XOD inhibitory effects of *A. aegerita*. Therefore, XOD suppression might be a potential mechanism underlying hypouricemia actions. SUA homeostasis is the consequence of uric acid excretion directly effectuated by renal transporters, of which, URAT1 located on the apical surface on nephron cells facilitate the UUA reabsorption into blood (Enomoto et al., 2002). The reduced level of URAT1 correlates with the decline in SUA. However, in the present study, AAE and AAW upregulated URAT1. Therefore, targeting URAT1 might not be a mechanism underlying the hypouricemia effects. On the other hand, OAT1 and URAT1 were also examined by Western blot and the levels were found to be elevated. Conventionally, OAT1 functions for uric acid secretion from blood to urine, such that its increase results in a decreased level of SUA. Thus, OAT1 might be a target of *A. aegerita*. In agreement with ELISA, URAT1 would not be the target of hypouricemia activity of *A. aegerita*. Comprehensively, we concluded that AAE and AAW reduced the level of SUA by suppressing XOD and elevating OAT1 rather than targeting URAT1.

BUN and creatinine are indexes for renal function (Kirtane et al., 2005). Compared to the normal group, administration of large amounts of PO in the hyperuricemia control induced a significant increase in BUN levels, suggesting some damage to the kidney. In contrast, allopurinol alleviated the slight impairment of the renal function of hyperuricemia mice, as compared to the hyperuricemia control. AAE and AAW reversed the damaged renal functions of hyperuricemia mice. Increased serum creatinine levels in the hyperuricemia control suggested a slight impairment of renal functions by large amounts of PO, which was consistent with that of the results of the BUN. Conversely, AAE and AAW might reverse the impeded renal functions of hyperuricemia mice, which was similar to that of the BUN. In addition to BUN and creatinine, organ weights are utilized for the conventional evaluation of toxicity (Michael et al., 2007). The current results showed that allopurinol negatively influenced the renal function. Nonetheless, AAE and AAW did not demonstrate such an effect. As the largest peripheral immune organ, spleen might reflect the current immune status. In hyperuricemia control, large amounts of PO induced slight inflammation as expected, which was alleviated by AAE and AAW, indicating critical anti-inflammatory activities. The toxicity assessment suggested that *A. aegerita*, as a routine consumable food, was not only non-toxic for inner organs but also beneficial to renal function and body inflammation.

In order to predict the constituents for hypouricemia action, we performed molecular docking screening (Wu et al., 2013) using our in-house compound database for *A. aegerita* and the crystallography structure of XOD since *A. aegerita* mainly interacted with XOD. Four compounds were screened out with good binding energy. From the docking, the four compounds were sited at the active cavity of XOD, which colonized the tunnel of the substrate and then evaded the entrance of xanthine. To confirm the activity of the screened compounds, HHC was selected since its availability commercially. The assaying result suggested the significant inhibitory activity of HHC on XOD. In order to assess the oral bioavailability of the four compounds, HIA, BBB, and CYP2D6 inhibition were forecasted (Meraj et al., 2012). These compounds were predicted to present good ADME properties. Also, the similarity to the drugs and Ames prediction support their non-mutagenic characteristics. Thus, the four compounds were bioavailable *in vivo*.

In summary, the AAE and AAW extracts of *A. aegerita* were prepared, and they produced excellent hypouricemia actions in hyperuricemia mice, rendering the level of SUA to decline to normal control. The hypouricemia activities might be attributed to their suppression on XOD activity and elevation for OAT1 levels rather than targeting URAT1. Also, only a slight negative impact was observed on inner organ functions. Molecular docking screening using our in-home database for *A. aegerita* was performed, and four compounds (2-formyl-3,5-dihydroxybenzyl acetate, 2,4-dihydroxy-6-methylbenzaldehyde, 2-(6-hydroxy-1H-indol-3-yl)acetamide, and 6-hydroxy-1H-indole-3-carbaldehyde) were identified as potential active compounds. Their inhibitory mechanism on XOD may be attributed to the placement of these molecules at the active cavity site of XOD, which might colonize the tunnel of the substrate to avoid the entrance of xanthine. Amongst, 6-hydroxy-1H-indole-3-carbaldehyde was tested and exhibited XOD inhibitory activity. Also, satisfactory intestinal absorption, good or optimal solubility, and low BBB index was predicted, thereby necessitating further study.

## Author contributions

TY was responsible for the concept and design of the study. TY performed the experiments and wrote the manuscript. SC, YX, OS, XL, DC, JS, CJ, and YL conducted a part of the experiments. All authors participated in the preparation of the manuscript and approved the final version.

### Conflict of interest statement

The authors declare that the research was conducted in the absence of any commercial or financial relationships that could be construed as a potential conflict of interest.
