# Supplementary material for: Hypouricemic Effects of Extracts From Agrocybe aegerita on Hyperuricemia Mice and Virtual Prediction of Bioactives by Molecular Docking
Source: Front Pharmacol. 2018 May 15;9:498. doi: 10.3389/fphar.2018.00498 (PMC5962791; doi:10.3389/fphar.2018.00498)
Supplement: Supplementary file 1 [file Table_1.DOC]

**Frontiers in Pharmacology**

Supplementary Materials

Hypouricemic Effects of Extracts from *Agrocybe aegerita* on Hyperuricemic Mice and Virtual Prediction of Bioactives by Molecular Docking

Tianqiao Yong ^a, b^, *, Shaodan Chen ^a, b^, Yizhen Xie ^a,b^, Ou Shuai ^a,b^, Xiangmin Li ^a, b^, Diling Chen ^a, b^, Jiyan Su ^a, b^, Chunwei Jiao ^a, b^, Yalei Liang ^a, b^

^a^ State Key Laboratory of Applied Microbiology Southern China, Guangdong Provincial Key Laboratory of Microbial Culture Collection and Application and Guangdong Open Laboratory of Applied Microbiology, Guangdong Institute of Microbiology, Guangzhou 510070, China

^b^ Guangdong Yuewei Edible Fungi Technology Co., Guangzhou 510663, China

***Corresponding author:**

Tianqiao Yong, Tel: +86-20-32059602, Fax: +86-20-32059602, E-mail: tianqiao@mail.ustc.edu.cn

**The following is included as additional supporting materials for this paper.**

**Captions:**

Page S4 **Fig. S1** The fingerprints of AAE and AAW and the HPLC chromatogram of the standard chemical (ergosterol) for *A. aegerita* identification. HPLC conditions-column: Waters Atlantis T3 RP-C18 column, 5μm, 250 mm × 4.6 mm; the mobile phases: methanol (2-100 % in 65 min) and water (98-0 % in 65 min), flowing rate: 0.8 ml/min; detection wavelength: 260 nm; temperature: 25 ^o^C; injection: 20μl. The retention times of the three peaks belonging to ergosterol were identical and overlapped well.

Page S4 **Fig. S2** The zoomed-in figure centered at 46.77 min of the fingerprints of AAE and AAW and the HPLC chromatogram of the standard chemical (ergosterol) for *A. aegerita* identification. The retention times of the three peaks belonging to ergosterol were identical and overlapped well.

**Experimental**

1. *HPLC conditions*

Chromatography was performed on a reversed-phase column (Waters Atlantis T3 RP-C18 column, 5μm, 250 mm × 4.6 mm) with a flow rate of 0.8 ml/min. Separation was carried out by linear gradient elution with methanol (5-100 % in 65 min) and water (95-0 % in 65 min). The detection wavelength was set at 260 nm, and the column temperature was kept at 25 ^o^C. The loading volume was 20 μl.

**Fig. S1**

**Fig. S2**
